# Overexpression of Global Regulator PbrlaeA Leads to the Discovery of New Polyketide in Fungus *Penicillium Brocae* HDN-12-143

**DOI:** 10.3389/fchem.2020.00270

**Published:** 2020-04-21

**Authors:** Lu Wang, Xianyan Zhang, Kaijin Zhang, Xiaomin Zhang, Tianjiao Zhu, Qian Che, Guojian Zhang, Dehai Li

**Affiliations:** ^1^Key Laboratory of Marine Drugs, Chinese Ministry of Education, School of Medicine and Pharmacy, Ocean University of China, Qingdao, China; ^2^Laboratory for Marine Drugs and Bioproducts, Pilot National Laboratory for Marine Science and Technology, Qingdao, China

**Keywords:** genome mining, *Penicillium brocae*, overexpression, global regulator PbrlaeA, silent gene cluster, polyketide, fumigatin chlorohydrin

## Abstract

Overexpression of the PbrlaeA gene of the fungus *Penicillium brocae* HDN-12-143 resulted in the isolation of four compounds including fumigatin chlorohydrin (**1**), whose configuration has not been reported before, and one new compound iso-fumigatin chlorohydrin (**2**). All structures including absolute configurations were elucidated on the basis of comprehensive spectroscopic data, ^13^C NMR calculations, and ECD calculations. Compounds **1** and **2** exhibited cytotoxic activity against HL-60 with IC_50_ of 18.63 and 24.83 μM.

## Introduction

LaeA is a broad-domain factor that can regulate the production of secondary metabolites (Keller et al., 2006; Kosalkova et al., 2009; Sarikaya et al., 2010). Overexpression of LaeA can enhance, or activate, the expression of gene clusters and produce new secondary metabolites (Jiang et al., 2016). During our recent work within the scope of exploring chemical diversity of fungal strains using tools manipulating the LaeA factor, the fungus *Penicillium brocae* HDN-12-143, isolated from sediment collected in the South China Sea, was selected as a candidate host to overexpress the LaeA gene due to its simple secondary metabolites background in comparison with its rich biosynthetic gene clusters, defined on the basis of bioinformatic analysis from the genome sequence, which shows a promising potential for secondary metabolite production (Figure S2).

For a start, we retrieved a native global regulator LaeA in *Penicillium brocae* 12-143 (PbrLaeA) by using LocalBLAST and InterPro analysis. Further overexpression of PbrLaeA in *Penicillium brocae* 12-143 led to the isolation of four compounds, including fumigatin chlorohydrin (**1**), whose configuration has not been reported before, as well as a new compound—iso-fumigatin chlorohydrin (**2**). Herein, we will report the biosynthetic pathway activation by overexpression of global regulator PbrLaeA, the isolation and characterization of resulting compounds, as well as the biological activity evaluation results.

## Materials and Methods

### General Experimental Procedures

The wild type and mutant strains of *Penicillium brocae* HDN12-143 were cultured on Difco™ Potato Dextrose Agar (Becton, Dickinson and Company, Sparks, USA). Full genome sequencing and assembly of HDN12-143 was manipulated by the Beijing Genomics Institute. PCR amplifications and verifications were performed on a T100™ Thermal Cycler (Bio-Rad Laboratories Inc., Singapore). Agarose electrophoresis was conducted on DYY-6C Type Electrophresis Apparatus (Liuyi Biotechnology, Beijing, China) and analyzed by SensiAnsys (Peiqing Science & Technology, Shanghai, China). UV spectra were recorded on Beckman DU 640 spectrophotometer (Beckman Coulter Inc., Brea, CA, USA). IR spectra were taken on Bruker tensor-27 spectrophotometer in KBr discs (Bruker Corporation, Billerica, MA, USA). Specific rotations were measured on JASCO P-1020 digital polarimeter (JASCO Corporation, Tokyo, Japan). ESIMS were obtained on Thermo Scientific LTQ Orbitrap XL mass spectrometer (Thermo Fisher Scientific, Waltham, MA, USA) or Micromass Q-TOF ULTIMA GLOBAL GAA076 LC Mass spectrometer (Wasters Corporation, Milford, MA, USA). CD spectra were measured on JASCO J-715 spectropolarimeter (JASCO Corporation, Tokyo, Japan). NMR spectra were recorded on Agilent 500 MHz DD2 spectrometer using TMS as internal standard, and chemical shifts were recorded as δ-values (Agilent Technologies Inc., Santa Clara, CA, USA). Semi-preparative HPLC was performed on an ODS column [HPLC (YMC-Pack ODS-A, 10 × 250 mm, 5 μm, 3 mL/min)] (YMC Co., Ltd., Kyoto, Japan). Medium-pressure preparation liquid chromatography (MPLC) was performed on a Bona-Agela CHEETAHTM HP100 (Beijing Agela Technologies Co., Ltd., Beijing, China). Column chromatography (CC) was performed with silica gel (200–300 mesh, Qingdao Marine Chemical Inc., Qingdao, China), and Sephadex LH-20 (Amersham Biosciences, San Francisco, CA, USA), respectively.

### Isolation and Identification of Fungal Material

The wild type fungus HDN-12-143 was isolated from a sediment sample collected in the South China Sea (China). The strain was incubated in potato dextrose agar medium at 28°C for 4 days, following which, it was incubated using the CTAB method (Tang et al., 2017) to obtain the genomic DNA library. By using classical microscopic analysis and ITS sequence alignment, HDN-12-143 was identified as *Penicillium brocae* (GenBank accession number MN410885). *P. brocae* HDN-12-143 was deposited at the Key Laboratory of Marine Drugs, the Ministry of Education of China, School of Medicine and Pharmacy, Ocean University of China, Qingdao, People's Republic of China.

### Genome Mining

The genomic DNA of *Penicillium brocae* HDN12-143 was analyzed on antiSMASH (https://fungismash.secondarymetabolites.org/). Following this, the sequence of *PbrlaeA* was identified by Localblast (Altschul et al., 1997) and analyzed with the reported LaeA obtained in NCBI (https://www.ncbi.nlm.nih.gov/). For the evolutionary relationship analysis, the amino acid sequences of PbrlaeA and other LaeA homologs from different *penicillium* species obtained from NCBI were aligned using the ClustalW (http://www.clustal.org/). The phylogenetic tree was constructed via the MEGA7 software (http://www.megasoftware.net/). The conserved domain of the PbrlaeA protein was scanned by using InterProScan tool (https://www.ebi.ac.uk/interpro/search/sequence-search).

### Construction of the PbrlaeA Expression Vector

The integrative vector pHyg, which mainly contains a constitutive promoter gpdA, ampicillin and hygromycin resistant genes, was digested with restriction endonucleases *Kpn*I and *Xba*I (New England Biolabs, NEB). The *PbrlaeA* gene was amplified from the genomic DNA library of the *P. brocae* HDN-12-143 using specific primers containing *Kpn*I and *Spe*I restriction sites (Table S1) via PCR catalyzed by *TransStart*^®^ *Fastpfu* DNA Polymerase (Transgen Biotech, Beijing, China). The PCR products were confirmed as correct using PCR analysis catalyzed by *2*×*EasyTaq*^®^ *PCR SuperMix* (Transgen Biotech, Beijing, China). After being digested with the above-mentioned corresponding endonucleases, the *PbrlaeA* gene was placed downstream of gpdA promoter within the pHyg vector to generate pHyg-PbrlaeA (Figure S4). The recombinant vector was transformed into *E. coli* Trans1-T1 competent cell to amplify plasmids for transformation.

### Transformation and Overexpression of *PbrlaeA*

The strain *P. brocae* HDN-12-143 was inoculated on PDA plates and cultured at 28°C for 4 days to grow fresh spores. Collected mycelium into 50 mL Tween buffer (50 μL Tween20 into 50 mL ddH_2_O) and spores are isolated by using 40 μm cell strainer (Solarbio Science & Technology, Bejing, China). Fresh spores were added into 50 mL PDB + YE medium (20% potato, 2% dextrose, and 0.4% yeast extract) in 250 mL Erlenmeyer flasks and germinated at 28 °C and 180 rpm for about 8 h. The germinated spores were collected by centrifugation at 4,000 rpm for 15 min, washed by 20 mL osmotic buffer (1.2 M MgSO_4_, 10 mM sodium phosphate, pH 5.8) twice, following which the germinated spores were suspended into 10 mL of osmotic buffer containing 30 mg lysing enzymes from *Trichodema harzianum* (SIGMA-ALDRICH, USA) and 20 mg Yatalase (TaKaRa, Japan), transferred into an empty sterile bottle and cultured in a shaker of 28°C at 80 rpm for 6 h to form protoplast. After enzymolysis, the mixture was transferred into a centrifuge tube and covered with a isopyknic protoplast trapping buffer (0.6 M sorbitol, 0.1 M pH 7.0 Tris-HCl) softly. After centrifugation at 4,000 rpm for 15 min at 4°C, protoplasts were collected in the interface of the above two buffers. Following this, the protoplasts were collected and washed by 20 mL STC buffer (1.2 M sorbitol, 10 mM CaCl_2_, 10 mM pH 7.5 Tris-HCl), and then resuspended in 2 mL STC buffer for subsequent transformation.

The pHyg-PbrlaeA was dissolved in 50 μL STC buffer after amplification, extraction and freeze-drying. Fungal transformation was carried out by mixing 100 μL of protoplasts and 50 μL of pHyg-PbrlaeA solution; the mixture was gently mixed with a pipet tip and incubated for 60 min on ice. Next, 600 μL of PEG solution (60% PEG, 50 mM calcium chloride and 50 mM pH 7.5 Tris-HCl) was added to the mixture, following which, the mixture was incubated at room temperature for 25 min. The mixture was gently spread on the regeneration PSA (PDA medium with 1.2 M sorbitol and 200 μg/mL hygromycin) medium and incubated at 28°C for 3 days (Ohashi et al., 2017).

### Transformants Screening

The hygromycin-resistant regenerated strains were transferred onto new PDA plates with 200 μg/mL hygromycin, respectively, for the second screening. The colonies that could grow were recognized as putative mutants.

The putative OE::PbrlaeA mutants and the wild-type strain were cultured on PDA media for 4 days at 28°C in an incubator for further genomic DNA extraction. PCR analysis to verify the gene insertion was carried out using two pairs of primers, as shown in Table S1 and Figure S5 (primers gpda-1 and YZ-LaeA-F to verify the upstream of the *PbrlaeA* gene, primers gpda-2 and YZ-LaeA-R to verify the downstream of the *PbrlaeA* gene). Following this screening, one transformant was recognized as the desired mutant.

### Fermentation and Extraction

The OE::PbrlaeA mutant strain was cultured under rotary shaker condition at 180 rpm at 28°C in 500 mL Erlenmeyer flasks containing 120 mL of liquid medium containing glucose (2%) and potato (20%) dissolved in naturally collected seawater (Huiquan Bay, Yellow Sea, Qiangdao, China). After 9 days shaker, the whole broth (30 L) was filtered through a cheesecloth to separate supernatant and mycelia. The former was extracted three times with EtOAc (3 × 30 L), while the latter was extracted three times with methanol (3 × 10 L) and concentrated under reduced pressure to afford an aqueous solution, which was then extracted three times with EtOAc (3 × 5 L). All EtOAc solutions were combined and concentrated under reduced pressure to obtain the extract (10 g).

### Purification

The extract was applied to a silica gel (300–400 mesh) column and was separated into 12 fractions (fraction 1 to fraction 12) with a step gradient elution of CH_2_Cl_2_-MeOH. Following this, fraction 5 was purified by MPLC, giving nine subfractions (fraction 5–1 to fraction 5–9). Fractions 5–6 were applied on semipreparative HPLC (20:80 MeOH–H_2_O, 3 mL/min) to afford compound **1** (8 mg) and compound **2** (20 mg). Fractions 5–9 were further purified by semipreparative HPLC (25:75 MeOH–H_2_O, 3 mL/min) to yield compound **3** (8 mg). Then, fraction 9 was separated and purified by MPLC to obtain nine subfractions (fractions 9–1 to fractions 9–9). Fractions 9–6 were applied on semipreparative HPLC (20:80 MeOH–H_2_O to 100:0 MeOH–H_2_O) to get compound **4** (8 mg).

### Computation Section

Conformational searches were performed, employing the systematic procedure implemented in Spartan'14 using the MMFF (Merck molecular force field). All MMFF minima were reoptimized with DFT calculations at the B3LYP/6-31+G(d) level using the Gaussian09 program (Frisch et al., 2010). The geometry was optimized starting from various initial conformations, with vibrational frequency calculations confirming the presence of minima. Time-dependent DFT calculations were performed on lowest-energy conformations (>5% population) for each configuration using 20 excited states and using a polarizable continuum model for MeOH. ECD spectra were generated using the program SpecDis (Bruhn et al., 2011) by applying a Gaussian band shape with a 0.30 eV width and 10 blue shifts to facilitate comparison to the experimental data. The ^13^C NMR chemical shifts of compounds **1** and **2** were calculated with the GIAO method at the B3LYP/6-311G (2d, p) levels in the Gaussian09 program (Frisch et al., 2010).

### Cytotoxicity Assay

The cytotoxicity assays were evaluated by the MTT method against the K562 and HL-60 cancer cell lines, and the SRB method against the H975, MGC803, and HO-8910 cancer cell lines. All of the biological evaluations were carried out as previously reported in the References (Yu et al., 2016; Gao et al., 2018).

## Results and Discussion

### Genome Mining and Overexpression of PbrlaeA

A LaeA analog named PbrlaeA (GeneBank No. MN410885) was identified via Localblast using AnlaeA (Q6TLK5.1) from *Aspergillus nidulans* as a query. The total size of the *PbrlaeA* gene is 1291 bp and the predicted open reading frame (ORF) is 1,122 bp, which may encode a polypeptide of 373 amino acids. Protein sequence alignment and phylogenetic analysis indicated that PbrlaeA was an S-adenosyl-L-methionine-dependent methyltransferase and closely related to PblaeA from *Penicillium brasilianum* (Figure S3).

**Figure 1:**
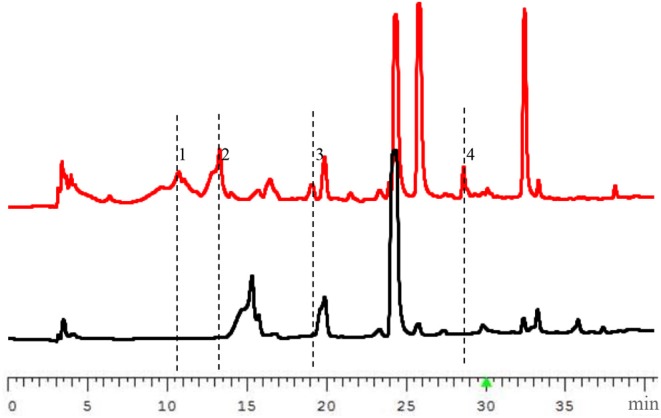
HPLC profiles of the extracts from wild type (WT, the lower diagram) and OE::PbrlaeA (MT, the upper diagram) strains of *P. brocae* HDN-12-143 (220 nm).

The PbrlaeA gene fragments added by *Kpn*I and *Spe*I (isocaudamer of *Xba*I) were amplified from genomic DNA of the strain *P. brocae* HDN-12-143 by using specific primers (Table S1) and ligated into the vector pHyg using restriction sites *Kpn*I and *Xba*I. The recombinant plasmid was transformed into *P. brocae* HDN-12-143 and screened with 200 μg/mL hygromycin in PDA medium. The mutant strain was further verified by diagnostic PCR (Figure S5). After being fermented with PDB medium under shaking condition at 28°C for 9 days, the high performance liquid chromatography (HPLC) analysis of the extract of the OE::PbrlaeA mutant strain showed a series of new peaks compared with the extract of the wild type *P. brocae* HDN-12-143 strain (Figure 1), which indicated the production of new secondary metabolites.

### Fermentation and Chemical Study

To confirm the structures of these newly produced secondary metabolites, the OE::PbrlaeA mutant strain was scaled up cultured (30 L) under rotary shaker condition at 180 rpm at 28°C. The extract (10 g) was fractionated by column chromatography, including silica gel, MPLC and semipreparative HPLC, leading to the isolation of compounds **1**–**4**.

**Figure 2:**
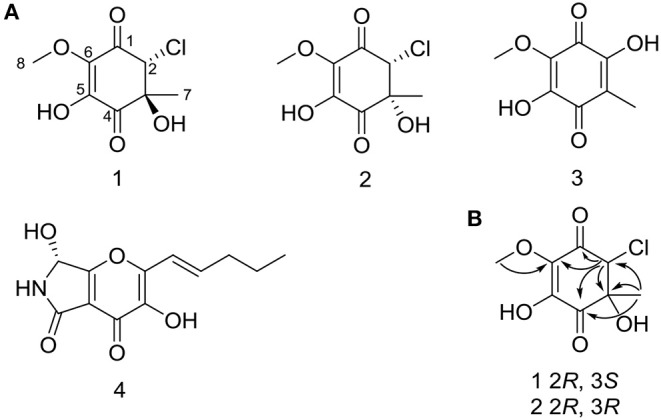
**(A)** Structures of **1**–**4** from mutant strain OE::PbrLaeA *P. brocae* HDN-12-143. **(B)** Key HMBC correlations of **1** and **2**.

**Figure 3:**
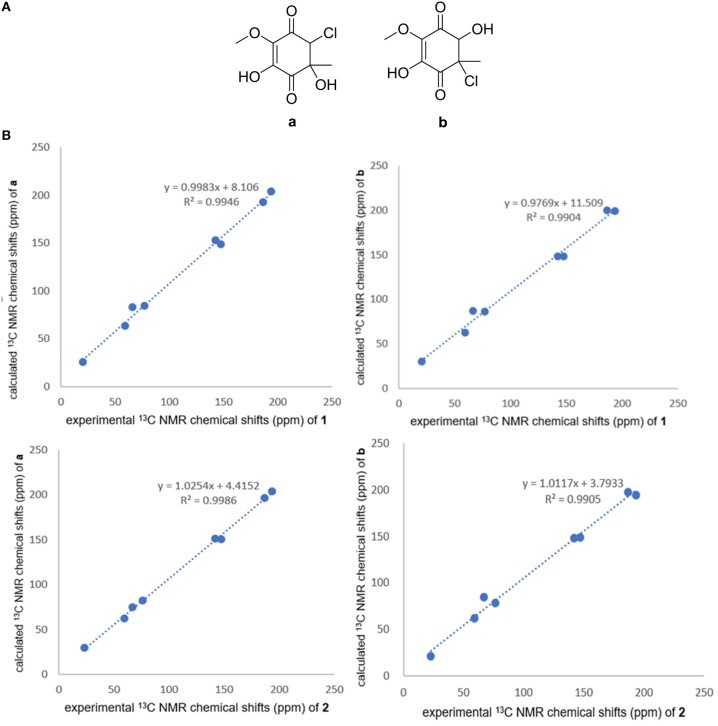
**(A)** Structures of **a** and **b**. **(B)** Correlation plots of experimental ^13^C NMR chemical shifts of **1** and **2** versus the corresponding calculated data for the proposed structures **a** and **b**.

Compounds **1** and **2** were obtained as yellowish oil. The molecular formula of **1** and **2** were all established to be C_8_H_9_O_5_Cl deduced by the [M – H]^−^ ion at m/z 219.0072 and 219.0071 (calcd for C_8_H_8_O_5_Cl: 219.0066) in the HRESIMS. The ^1^H NMR and ^13^C NMR spectroscopic data of compound **1** displayed one methyl, one oxymethyl, one methine, five quaternary carbons including two sp^2^ quaternary carbons and two ketone carbonyls. Based on the HMBC correlations from H_1_-2 to C-1, C-6, C-3, and C-4, from H_3_-7 to C-2, C-3, and C-4, and from H_3_-8 to C-6 (Figure 2), two possible planar structures **a** and **b**, where the locations of chlorine and hydroxyl groups were left undetermined (Figure 3A), were proposed for **1**. Further ^13^C NMR calculation method was adopted to confirm the accurate structure (Pierens, 2014). Calculation of the ^13^C NMR chemical shifts of **a** and **b** at the B3LYP/6-311G (2d, p) levels in CD_3_OD were obtained, and the experimental chemical shifts agreed well with the calculated data of **a** (*R*^2^ = 0.9946) (Figure 3B), assigning the planar structure of compound **1** as **a** (fumigatin chlorohydrin). Further comparing of the optical rotation data of **1** ${\left[[\alpha \right]}_{\text{D}}^{20}$ −102.6 (c 0.10, H_2_O) with that of fumigatin chlorohydrin ([α]_D_ −160 in H_2_O for fumigatin chlorohydrin] (Yamamoto et al., 1970) confirmed **1** as fumigatin chlorohydrin. The planar structure of fumigatin chlorohydrin was first reported in 1970 from the fungus *Aspergillus Fumigatus* (Yamamoto et al., 1970). However, the report only covered the ^1^H NMR data of fumigatin chlorohydrin, meanwhile the configuration and activity were not included. In the present work, we describe the absolute configuration for the first time.

**Figure 4:**
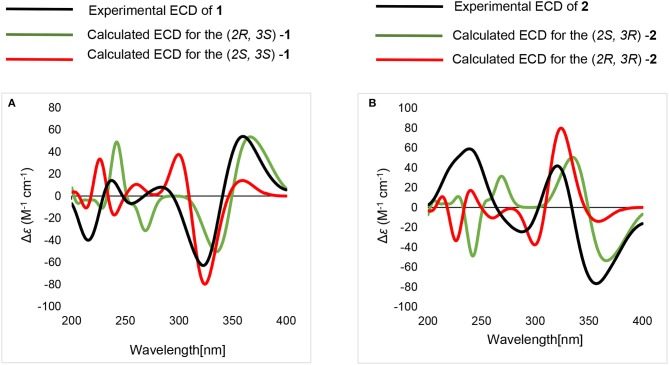
Calculated and experimental ECD spectra. **(A)** calculated and experimental ECD of (2*R*, 3*S*)-**1**, (2*S*, 3*S*)-**1**. **(B)** calculated and experimental ECD of (2*S*, 3*R*)-**2**, (2*R*, 3*R*)-**2**.

Compound **2** had the same planar structure as **1** based on NMR and calculation of the ^13^C NMR chemical shifts, while the minor variations in chemical shifts of C-2, C-3, and C-7 (δ_C_: 66.0, 76.6, and 20.2 in **1**; 67.0, 76.2, and 22.8 in **2**) revealed different configurations at the stereocenters. To establish the absolute configuration, the electronic circular dichroism (ECD) spectra of (2*R*, 3*S*), (2*R*, 3*R*), (2*S*, 3*R*), and (2*S*, 3*S*) were simulated using density functional theory (DFT) calculations performed at the B3LYP/6-31+G(d) level. The absolute configurations of (2*R*, 3*S*)-**1** and (2*R*, 3*R*)-**2** were unambiguously determined by the almost identical curves between the computational ECD curve (2*R*, 3*S*)-**1** and (2*R*, 3*R*)-**2** and the experimental one (Figure S1; Figure 4). Thus, compound **2** was confirmed to be a diastereoisomer of fumigatin chlorohydrin and named as iso-fumigatin chlorohydrin. From a biosynthetic view, fumigatin-like phenols are fundamentally derived from polyketide synthase biosynthetic (PKS) pathways, which adopted acetyl-CoA as precursor (Packter and Glover, 1965; Pacter, 1966). With regards to compounds **1**–**3**, the specific enzymes responsible for the tailoring steps, like oxidation and halogenation on the aromatic skeleton, remain an intriguing topic for further investigation.

#### Fumigatin Chlorohydrin (1)

${\left[\alpha \right]}_{\text{D}}^{20}$ −6.9 (c 0.10, MeOH); UV (MeOH) λ max (log ε) 232 (1.8), 310 (2.8) nm; IR (KBr) ν_max_ 3,417, 2,936, 1,716, 1,636, 1,457, 1,385, 1,328, 1,110, 1,029 cm^−1^; ^1^H NMR (CD_3_OD, 500 MHz) and ^13^C NMR (CD_3_OD, 125 MHz) data are shown in Table 1, HRESIMS m/z: 219.0072 [M – H]^−^ (calcd. for C_8_H_8_O_5_Cl: 219.0066).

**Table 1:** ^1^H NMR (500 MHz) and ^13^C NMR (125 MHz) Spectroscopic Data of **1** and **2** in CD_3_OD.

**Position**	**1**		**2**	
	*****δ***_**C**_**	*****δ***_**H**_**	*****δ***_**C**_**	*****δ***_**H**_**
1	186.0		186.5	
2	66.0	4.88, s	67.0	4.75, s
3	76.6		76.2	
4	193.4		193.1	
5	147.2		147.0	
6	142.3		141.9	
7	20.2	1.44, s	22.8	1.53, s
8	59.0	3.92, s	59.2	3.92, s

#### Iso-Fumigatin Chlorohydrin (2)

${\left[\alpha \right]}_{\text{D}}^{20}$ −15.3 (c 0.10, MeOH); UV (MeOH) λ max (log ε) 232 (1.8), 310 (2.8) nm; IR (KBr) ν_max_ 3,445, 2,952, 1,676, 1,625, 1,447, 1,358, 1,242, 1,054, 985, 960 cm^−1^; ^1^H NMR (CD_3_OD, 500 MHz) and ^13^C NMR (CD_3_OD, 125 MHz) data are shown in Table 1, HRESIMS m/z: 219.0071 [M–H]^−^ (calcd. for C_8_H_8_O_5_Cl: 219.0066).

The known compounds were identified as spinulosin (**3**) (Anslow and Raistrick, 1938) and pyranonigrin F (**4**) (Meng et al., 2015) through comparison of the NMR and MS data with the reported ones.

The MS, 1D and 2D NMR spectra for compounds **1**–**2** are available as Supplementary Material.

### Activity Assay

All the compounds were tested for their cytotoxicity against five cancer cell lines (HL-60, K562, H1975, MGC803 and HO-8910) (Yu et al., 2016). Compounds **1** and **2** exhibited weak activities against HL-60 with IC_50_ of 18.63 and 24.83 μM (Table S2). Besides, compounds **1** and **2** were also tested for the antimicrobial activity against *Bacillus subtilis* and antioxidant capacities by DPPH free radical scavenging assay, but no activity was observed (IC_50_ > 100 μM) (Yu et al., 2016; Gao et al., 2018).

## Conclusions

Four compounds were isolated from the fungus *Penicillium brocae* HDN-12-143 by overexpression of the LaeA family gene of PbrlaeA. Among them, the planar structure of **1** and **2** was determined by comprehensive spectroscopic data with ^13^C NMR calculations. The absolute configuration was determined by calculating the ECD. Compounds **1** and **2** exhibited weak cytotoxicity against HL-60 with IC_50_ of 18.63 and 24.83 μM. The above study showed that overexpression of the global regulator LaeA analogs could be an efficient method to activate the silent biosynthetic pathway of marine-derived *Penicillium* strains, generating chemical diversity in their secondary metabolites profile.

## Data Availability Statement

The datasets generated for this study can be found in the GeneBank No. MN410885.

## Author Contributions

The contributions of the respective authors are as follows: LW drafted the work and performed the fermentation, extraction, as well as the isolation. XianZ constructed the plasmids and was involved in the acquisition of mutant strains, performed the biological evaluations, and bioinformatic analysis. KZ was involved in the acquisition of mutant strains and bioinformatic analysis. XiaoZ performed the biological evaluations. TZ and QC contributed to checking and confirming all the procedures of the isolation and identification. GZ and DL designed the study, supervised the laboratory work, and contributed to the critical reading of the manuscript.

## Conflict of Interest

The authors declare that the research was conducted in the absence of any commercial or financial relationships that could be construed as a potential conflict of interest.
