# Supplementary material for: Overexpression of Global Regulator PbrlaeA Leads to the Discovery of New Polyketide in Fungus Penicillium Brocae HDN-12-143
Source: Front Chem. 2020 Apr 21;8:270. doi: 10.3389/fchem.2020.00270 (PMC7186497; doi:10.3389/fchem.2020.00270)
Supplement: Supplementary file 1 [file Data_Sheet_1.docx]

Supplementary Material

Overexpression of Global Regulator PbrlaeA Leads to the Discovery of New Polyketide in Fungus *Penicillium Brocae* HDN-12-143

Lu Wang^1†^, Xianyan Zhang^1†^, Kaijin Zhang^1^, Xiaomin Zhang^1^, Tianjiao Zhu^1^, Qian Che^1^, Guojian Zhang^1,2*^, Dehai Li^1,2*^

^1^ Key Laboratory of Marine Drugs, Chinese Ministry of Education, School of Medicine and Pharmacy, Ocean University of China, Qingdao, People’s Republic of China

^2^ Laboratory for Marine Drugs and Bioproducts, Pilot National Laboratory for Marine Science and Technology, Qingdao, People’s Republic of China

† These authors contributed equally to this work.

*** Correspondence:**

Guojian Zhang

zhangguojian@ouc.edu.cn;
Dehai Li
dehaili@ouc.edu.cn

**Table S1.** The primers used in this study.............................................................................................3

**Table S2**. The initial experimental data (OD Value and inhibition ratio%) of cytotoxicity assays.......4

**Figure S1**. Calculated and experimental ECD spectra of compounds **1** and **2**…………………………5

**Figure S2**. AntiSMASH analysis of the genome of the strain Penicillium brocae HDN 12-143……..................................................................................................................................................5

**Figure S3**. Phylogenetic tree analysis of PbrlaeA………............................................................... 6

**Figure S4.** Map of the vector pHyg backbone and the constructed plasmid of pHyg-PbrlaeA……..6

**Figure S5.** PCR analysis of gene insertion……………………………………………….…………..7

**Figure S6**. ^1^H NMR (500MHz, CD_3_OD) spectrum of **1** ............................................7

**Figure S7**. ^13^C NMR (125MHz, CD_3_OD) spectrum of **1** ...........................................8

**Figure S8**. HSQC spectrum of **1** in CD_3_OD...............................................................8

**Figure S9**. ^1^H-^1^H COSY spectrum of **1** in CD_3_OD ..................................................9

**Figure S10.** HMBC spectrum of **1** in CD_3_OD ............................................................9

**Figure S11**. HRESIMS spectrum of **1**........................................................................10

**Figure S12**. ^1^H NMR (500MHz, CD_3_OD) spectrum of **2** ..........................................10

**Figure S13**. ^13^C NMR (125MHz, CD_3_OD) spectrum of **2** .........................................11

**Figure S14**. HSQC spectrum of **2** in CD_3_OD.............................................................11

**Figure S15**. ^1^H-^1^H COSY spectrum of **2** in CD_3_OD .................................................12

**Figure S16.** HMBC spectrum of **2** in CD_3_OD ..........................................................12

**Figure S17**. HRESIMS spectrum of **2**........................................................................13

**Figure S18**.IR spectrum of **1**........................................................................13

**Figure S19**.IR spectrum of **2**........................................................................14

**Figure S20**. ^1^H NMR (400MHz, D_2_O) spectrum of **1** ..........................................14

**Table S1.** The primers used in this study. (5’ to 3’).

| Primers | Sequences |
| --- | --- |
| LaeA-F | GGactagtATGCCGCTCGACGATGATC |
| LaeA-R | CGGggtaccTTATAATTTGGTCAGCGGCTTGCG |
| gpda-1 | TACAGACAAGCTGTGACCGTCTC |
| gpda-2 | CGTTAAGTGGATCTCGGTGACGG |
| YZ-LaeA-F | ATCTGGTTAGCTCCATGGCCTC |
| YZ-LaeA-R | ACCGATCGAGACCCTTCAG |

Note: The sites of the restriction endonucleases are represented by lowercase letters.

**Table S2**. The initial experimental data (OD Value and inhibition ratio%) of cytotoxicity assays

|  | Concentration (*µ*M) | OD Value | inhibition ratio% |
| --- | --- | --- | --- |
| blank group |  | 1.72±0.05 |  |
| control group |  | 1.85±0.05 |  |
| Positive group (ADM) | 1 | 0.26±0.01 | 100.00 |
| **1** | 30 | 0.34±0.01 | 94.90 |
|  | 15 | 1.60±0.02 | 8.54 |
|  | 7.5 | 1.68±0.06 | 2.58 |
| **2** | 30 | 0.66±0.04 | 73.32 |
|  | 15 | 1.65±0.03 | 4.84 |
|  | 7.5 | 1.74±0.03 | -1.01 |

**Figure S1**. Calculated and experimental ECD spectra of compounds **1** and **2** (A) calculated and experimental ECD spectra of (2*R*, 3*S*)-**1**, (2*S*, 3*S*)-**1**. (B) calculated and experimental ECD spectra of (2*R*, 3*S*)-**1**, (2*S*, 3*S*)-**2**.

1. (B)


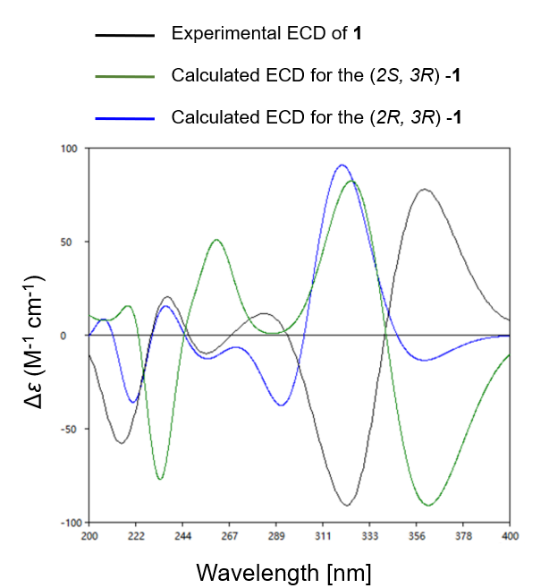

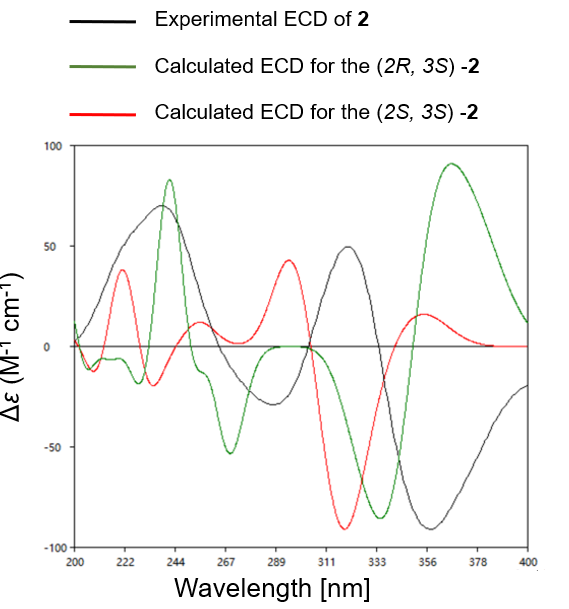


**Figure S2.** AntiSMASH analysis defined the biosynthetic pathways from *Penicillium brocae* HDN 12-143. The identified 39 secondary metabolite regions include 13 PKS, 13 NRPS, 6 Terpene, 1 Indole, 6 PKS-NRPS hybrids gene clusters.


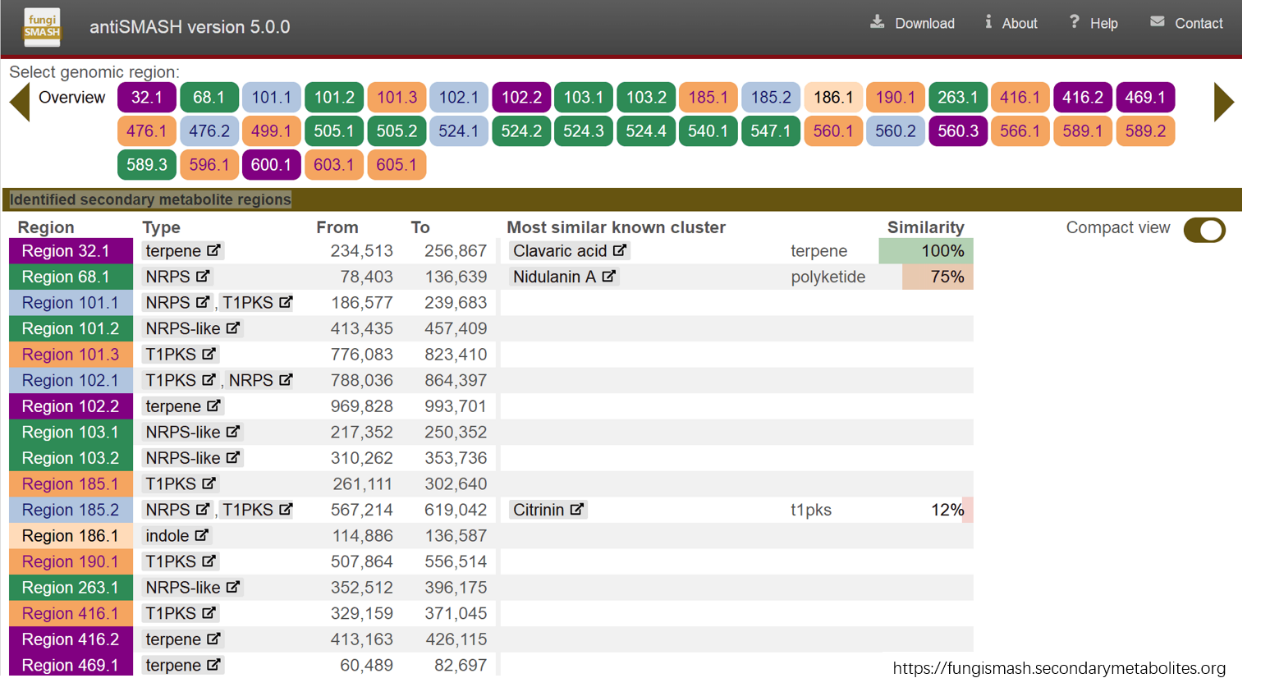


**Figure S3.** Phylogenetic analysis of PbrlaeA. The figure highlights the uniqueness of the PbrlaeA (in bold) among the ones reported in the NCBI database. Phylogenetic tree was generated by maximum likelihood method based on the Poisson correction model.


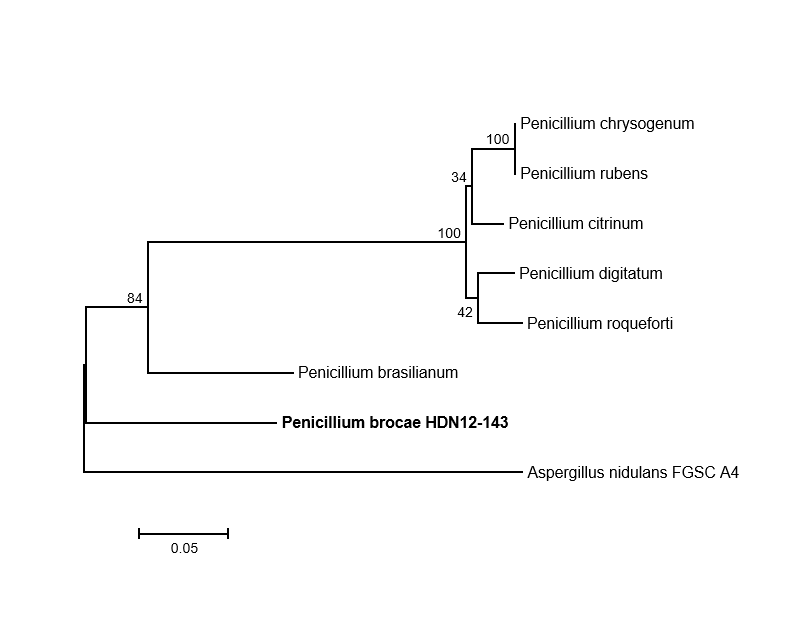


**Figure S4.** Map of the vector pHyg backbone and the constructed plasmid of pHyg-PbrlaeA.


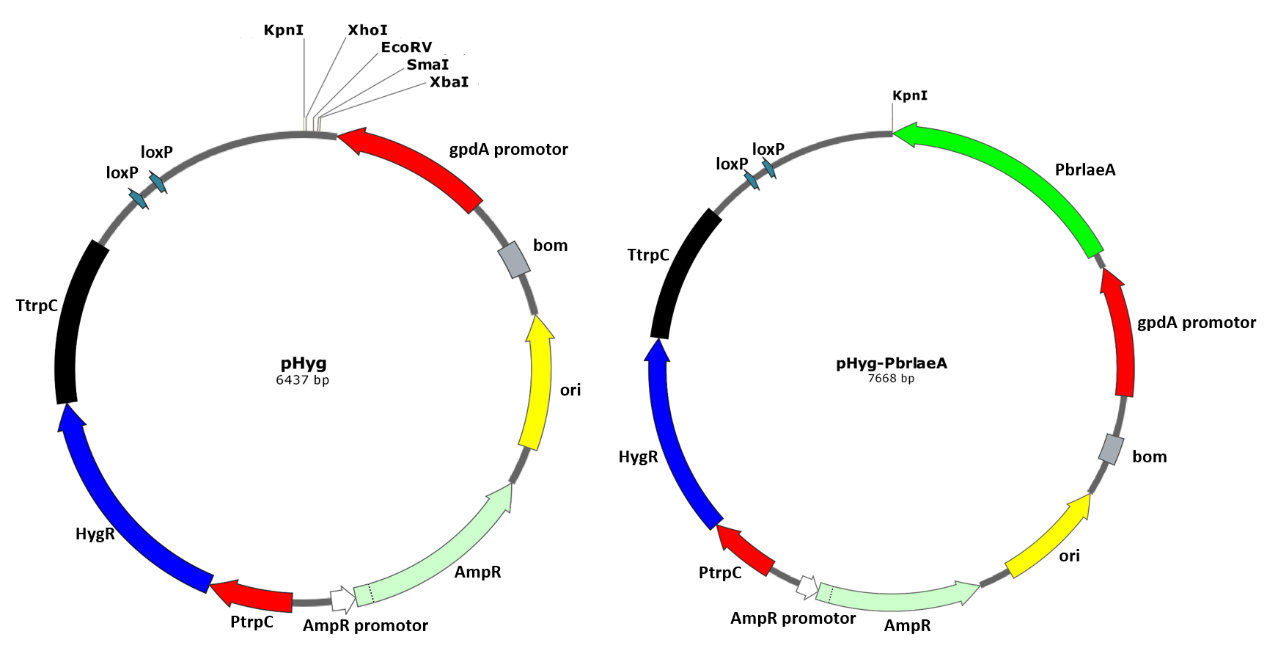


**Figure S5.** PCR analysis of gene insertion. The mutant strain HDN12-143-OE::LaeA showed correct sized products verified by PCR.


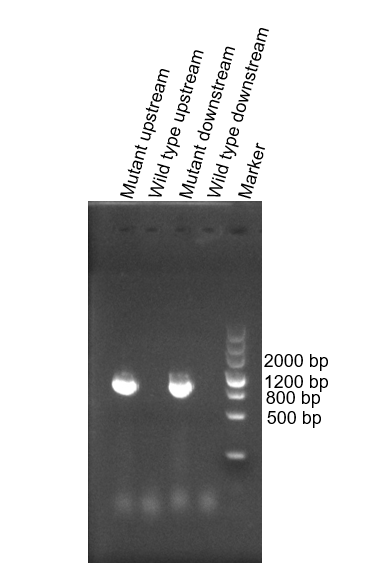


**Figure S6**. ^1^H NMR (500MHz, CD_3_OD) spectrum of **1**

**Figure S7**. ^13^C NMR (125MHz, CD_3_OD) spectrum of **1**

**Figure S8**. HSQC spectrum of **1** in CD_3_OD

**Figure S9**. ^1^H-^1^H COSY spectrum of **1** in CD_3_OD.

**Figure S10**. HMBC spectrum of **1** in CD_3_OD.

**Figure S11**. HRESIMS spectrum of **1**


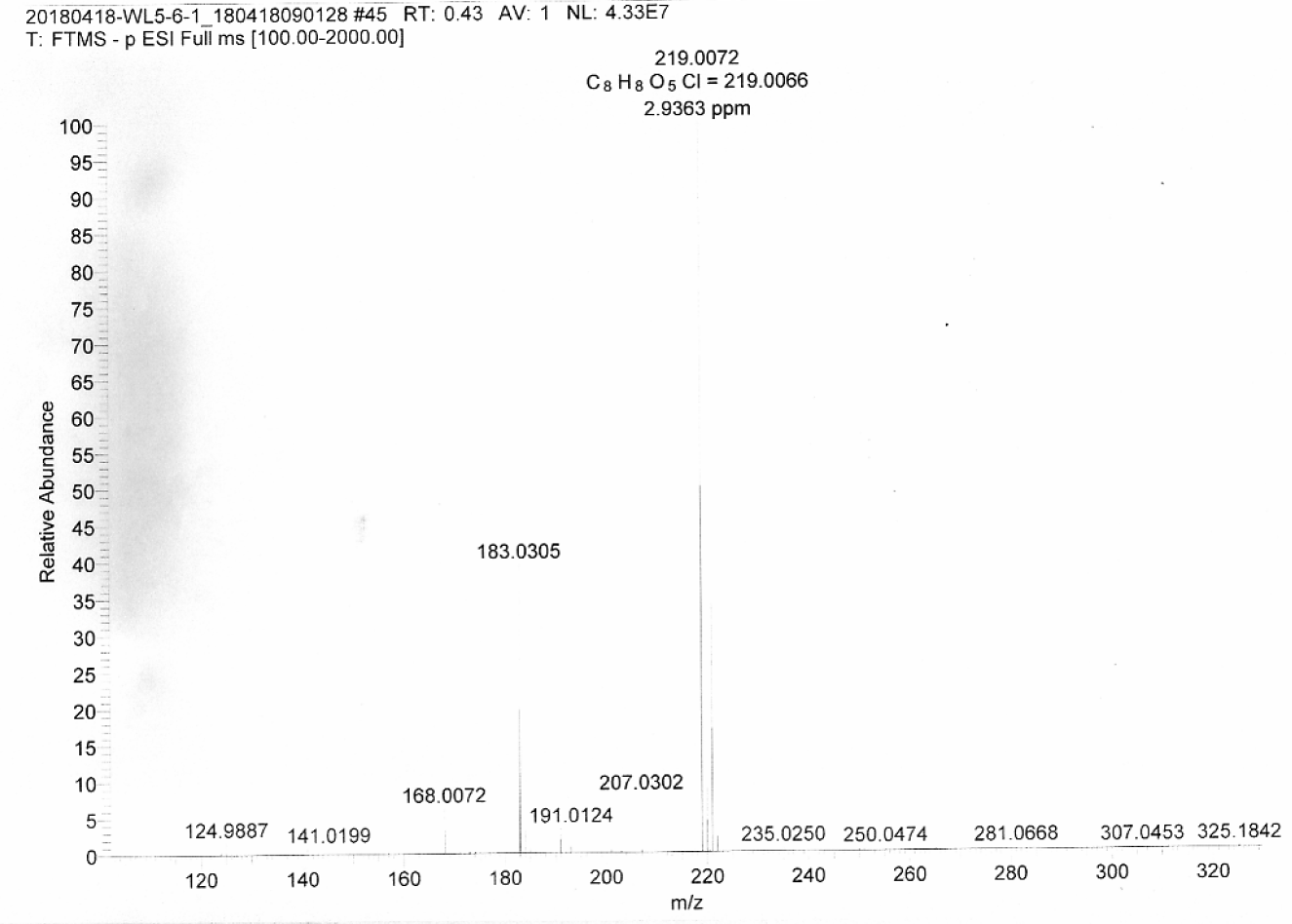


**Figure S12.** ^1^H NMR (500MHz, CD_3_OD) spectrum of **2.**

**Figure S13**. ^13^C NMR (125MHz, CD_3_OD) spectrum of **2.**

**Figure S14**. HSQC spectrum of **2** in CD_3_OD_._

**Figure S15**. ^1^H-^1^H COSY spectrum of **2** in CD_3_OD_._

**Figure S16**. HMBC spectrum of **2** in CD_3_OD.

**Figure S17**. HRESIMS spectrum of **2**


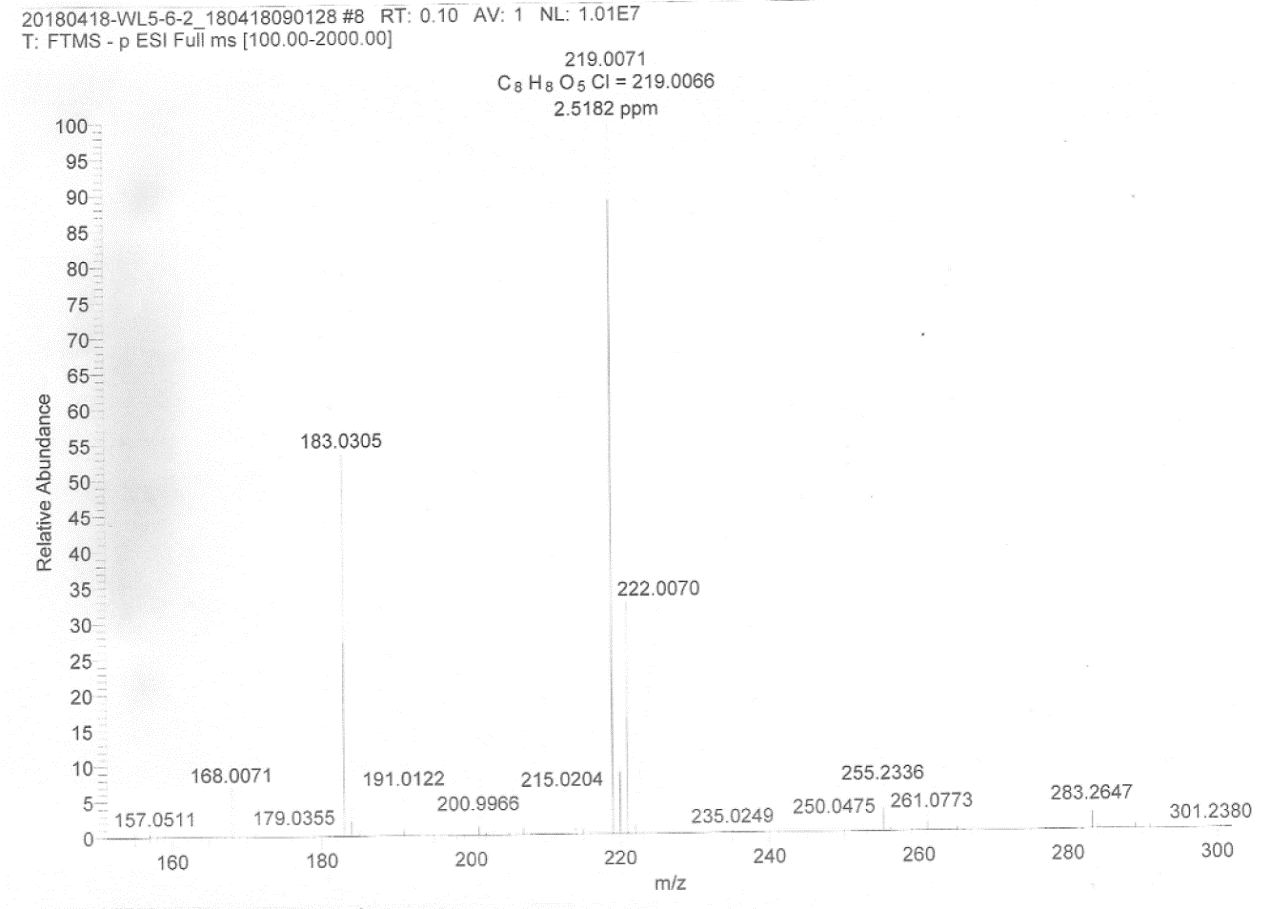


**Figure S18**. IR spectrum of **1**.


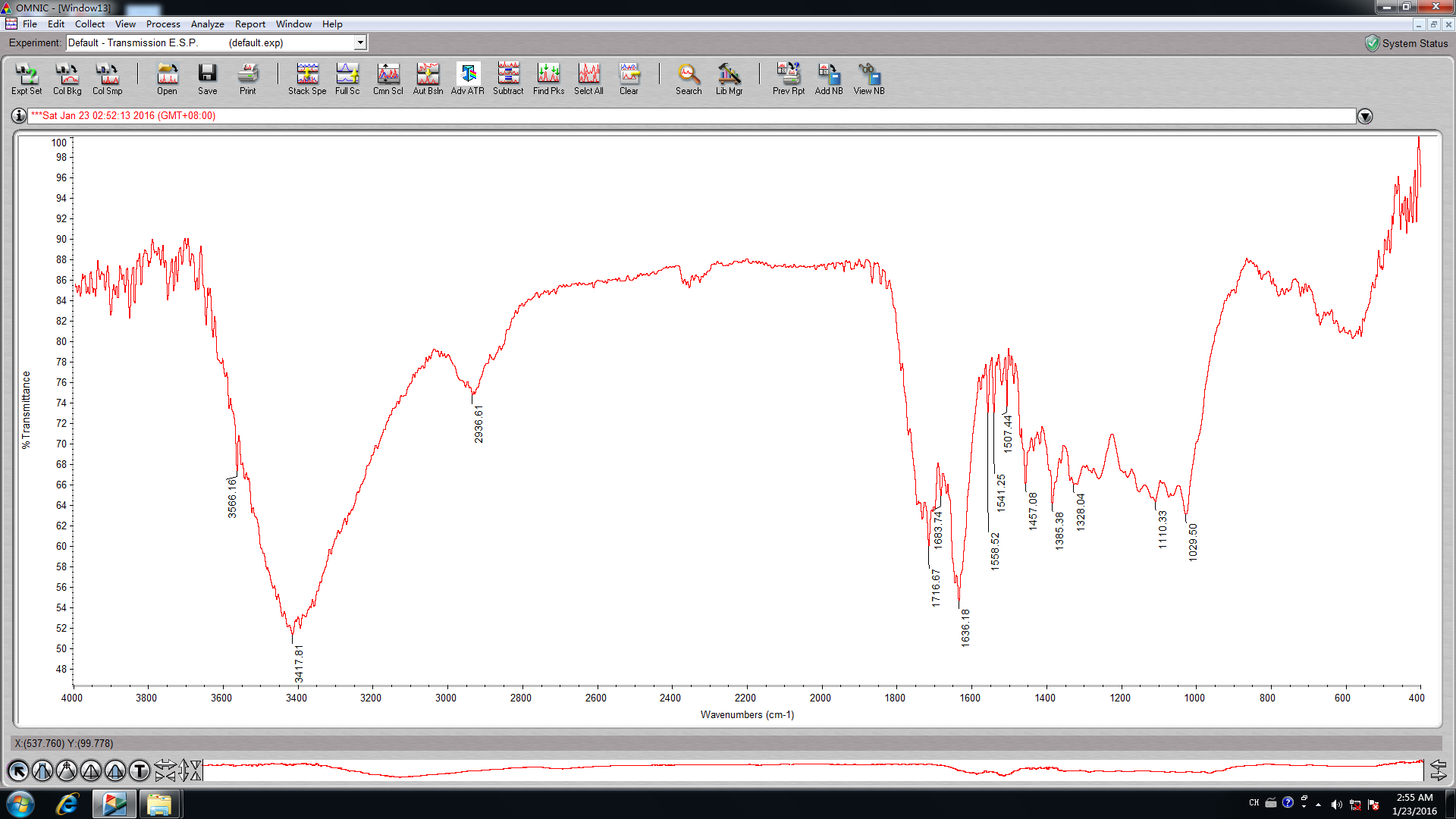


**Figure S19**. IR spectrum of **2**.


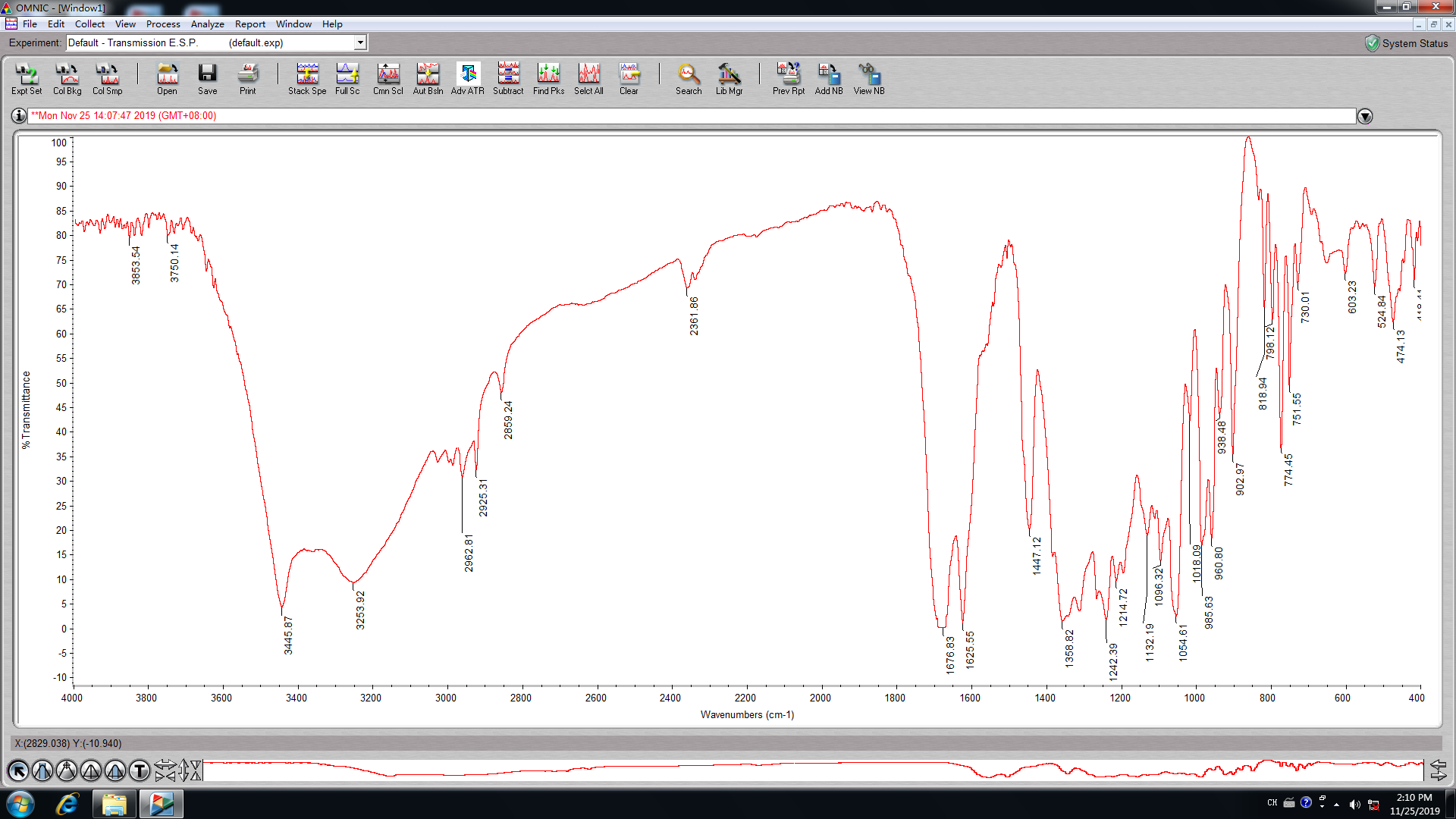


**Figure S20**. ^1^H NMR (400MHz, D_2_O) spectrum of **1**
